# Event and Comment

**Published:** 1919-08

**Authors:** 


### DENTAL REGISTER

THE MAGAZINE OF DENTISTRY

Vol. LXXIII AUGUST, 1919 No. 8

### EVENT AND COMMENT

**Something New in Dental Society Organization** The Indianapolis Dental Society has provided membership for persons who are affiliated in any way in carrying on dental practice, whether they are registered graduate dentists or not. Such persons are given affiliated or associate membership. They are known as associate members and have the privilege of attending all meetings of the Society and may participate in the discussions. They do not vote or hold office, but are encouraged to contribute to the proceedings of the meetings in such ways as they can or may wish to do. This permits the technical man or other office assistant or associate to attend and participate in any meeting where the proceedings may be of interest to him.

The idea seems to be to bring about more intelligent and sympathetic co-operation of the various persons doing dental work of any kind, with the idea uppermost that each member shall contribute from his experience something that may increase his own efficiency in practice and may lead to a uniform standardization of methods and the greater perfection of skill in all departments. The scheme is ideal and democratic, and in line with the general tendency of present-day movements in political and civic life, but we are wondering how long it will last, and what the outcome may be.

There certainly must be great differences in viewpoints, and such a change in the conduct of the dental society is liable to introduce complications in administration that may ultimately disrupt the organization, unless there is a strong social and professional spirit developed that will unify the differing aims and ideals into an entirely new form of association effort.

If properly carried out, it ought to result in a better understanding as to what real dental service should be, and the interchange of experiences ought to be of great benefit to those who have never had the benefit of a professional training. It should react on the dentist, also, in improving his own technical skill and in introducing more systematic and efficient business methods into his practice. It may be another symptom of the evolutionary trend of the times that will help to solve the serious problem of how we are to meet the dental demands of the future with a limited supply of professional graduates.

